# The tropomyosin 3.1/3.2 inhibitor ATM-3507 alters B-cell actin dynamics and impairs the growth and motility of diffuse large B-cell lymphoma cell lines

**DOI:** 10.3389/fimmu.2025.1668379

**Published:** 2025-11-05

**Authors:** Abhishek Bedi, Kate Choi, Alyssa Iskierski, Michael R. Gold

**Affiliations:** Department of Microbiology and Immunology and the Life Sciences Institute, University of British Columbia, Vancouver, BC, Canada

**Keywords:** tropomyosin 3.1/3.2 (Tpm3.1/3.2), B cell, diffuse large B-cell lymphoma (DLBCL), ATM-3507, actin cytoskeleton, B-cell antigen receptor (BCR), cell spreading, cell migration

## Abstract

**Introduction:**

By stabilizing actin filaments and recruiting non-muscle myosin II, the closely related tropomyosin (Tpm) isoforms Tpm3.1 and Tpm3.2 support actin-dependent processes including membrane dynamics, cell migration, and cytokinesis. Actin dynamics are essential for B cell function, but the roles of Tpm3.1 and 3.2 (collectively termed Tpm3.1/3.2) in B cells have not been explored. Moreover, new treatments are needed to limit the growth and dissemination of diffuse large B-cell lymphoma (DLBCL), the most prevalent B-cell malignancy.

**Methods:**

To test whether Tpm3.1/3.2 is essential for B-cell actin dynamics and could be a target for treating DLBCL, we employed ATM-3507, a compound that selectively interferes with Tpm3.1/3.2 function.

**Results:**

We show that ATM-3507 treatment inhibited B-cell receptor-induced formation of the peripheral ring of branched actin that drives cell spreading and also prevented the formation of actomyosin arcs at the inner face of the peripheral actin ring. Tpm3.1/3.2 localizes to these structures during B-cell spreading. Treating DLBCL cell lines with ATM-3507 inhibited cell growth and caused the cells to accumulate in the G2/M phase of the cell cycle. Furthermore, ATM-3507 markedly reduced CXCL12-stimulated chemotaxis and integrin-dependent motility of DLBCL cell lines on fibronectin.

**Conclusion:**

Tpm3.1/3.2 orchestrates key actin-driven processes in B cells, and drugs that target Tpm3.1/3.2 may be useful adjuncts for treating DLBCL.

## 1 Introduction

B cells contribute to both protective immunity and pathological autoimmunity by secreting antibodies (Abs) and cytokines, and by presenting antigens to T cells (1–5). Multiple aspects of B-cell function depend on remodeling of the actin cytoskeleton (6–8). Actin-dependent membrane protrusion is required for B-cell motility and chemotaxis, processes that underlie the trafficking of B cells into and within lymphoid organs and inflamed tissues (9–11). Within lymphoid organs, B cells can encounter soluble or cell-associated antigens that are recognized by their clonotypic B-cell receptor (BCR). When B cells bind antigens displayed on the surface of antigen-presenting cells (APCs) such as follicular dendritic cells, BCR-induced actin polymerization drives the formation of membrane protrusions that scan the APC surface for additional antigens, thereby increasing BCR signaling and promoting B-cell activation (12, 13). The roles of actin dynamics in optimizing BCR signaling, enabling cell motility, and mediating cell division are essential for normal B-cell function. Actin remodeling also supports the growth of B-cell lymphomas and their ability to spread from the lymph node site of origin to other organs (14).

Tropomyosins (Tpms) are proteins that regulate actin-dependent cellular processes by stabilizing actin filaments and by modulating their interactions with actin-binding proteins (15–17). Tpms have an elongated coiled-coil structure and form dimers that bind to 6–7 consecutive actin subunits within a filament. Head-to-tail interactions between adjacent Tpm dimers result in a continuous Tpm chain (or co-filament) that runs along the actin filament (18–20). In mammals, more than 40 Tpm isoforms are generated from the *Tpm1*, *Tpm2*, *Tpm3*, and *Tpm4* genes by alternative splicing (16). The distinct N- and C-terminal sequences of different Tpm isoforms favor the formation of Tpm homopolymers that associate with distinct segments of actin filaments and with different actin structures within the cell (21, 22). Because different Tpm isoforms recruit distinct sets of actin-binding proteins, Tpms specify the functions and turnover dynamics of the actin filaments that they decorate (17, 21, 23). For example, the Tpm3.1, Tpm3.2, Tpm4.2, and Tpm1.7 isoforms recruit myosin II to actin filaments, activate the myosin ATPase, and contribute to actomyosin-dependent processes including membrane protrusion, stress fiber formation, and cell migration (21, 24, 25).

The *Tpm3* gene is essential for embryonic development (26, 27), and the Tpm3.1 and Tpm3.2 splice variants are among the most extensively studied Tpm isoforms. Tpm3.1 and Tpm3.2 share an identical amino acid sequence except for several non-conservative amino acid changes in a central region of the protein that arise from alternate exon usage (28). They are often referred to collectively as Tpm3.1/3.2 because they are not distinguishable by existing Abs (25) and share a unique C-terminal pocket that binds the small molecule inhibitors TR-100 and ATM-3507 (29–31). These inhibitors bind to actin-associated Tpm3.1/3.2 polymers and ablate the ability of Tpm3.1/3.2 to stabilize actin filaments and recruit myosin (25, 29). Biochemical experiments with purified components demonstrated that Tpm3.1 stabilizes actin filaments by shielding them from the actin-severing protein cofilin and by recruiting the pointed-end capping protein tropomodulin, which prevents the dissociation of actin monomers (23, 32). Tpm3.1/Tpm3.2 have been implicated in multiple actomyosin-dependent processes (17), including cell motility and migration (33, 34), stress fiber formation (25), focal adhesion dynamics (33, 34), cell morphology (35, 36), exocytosis (37), insulin-mediated glucose uptake (38), and cell proliferation (39). Although *Tpm3* is expressed in mouse (http://rstats.immgen.org/Skyline/skyline.html) and human (https://singlecell.broadinstitute.org/single_cell/study/SCP345/ica-blood-mononuclear-cells-2-donors-2-sites?genes=TPM3) B lymphocytes, the role of Tpm3.1/3.2 in B cells has not been investigated.

Diffuse large B-cell lymphoma (DLBCL) is one of the most prevalent forms of hematologic malignancies (40). The current standard treatment regimen for DLBCL, R-CHOP, consists of the anti-CD20 monoclonal Ab rituximab, doxorubicin, vincristine, cyclophosphamide, and prednisone (41). Despite these treatments, ~40% of the patients experience relapse or have tumors that are refractory to these drugs (42, 43). Advances in genome sequencing and transcriptomics have identified driver mutations and altered signaling pathways that contribute to the survival and proliferation of DLBCL (44, 45). DLCBCL cells can be classified into two main types based on their cell of origin (46). The more aggressive activated B cell-like DLBCLs (ABC-DLBCL) are driven by chronic/activated BCR signaling and NF-κB activation (47). In contrast, germinal center B cell-like DLBCLs (GCB-DLBCL) require tonic/basal BCR signaling but are primarily driven by the overexpression of c-Myc or by the dysregulation of epigenetic regulators such as EZH2, KMT2D, EP300, EBF1, and IRF8 (44, 48, 49). Recent studies have identified an additional type of DLBCL, the germinal center dark zone signature subgroup, which exhibits a unique gene expression signature and has a poor prognosis, even after R-CHOP (50). Beyond R-CHOP, targeting BCR signaling with inhibitors of Btk, Syk, or the phosphoinositide 3-kinase/Akt/mTOR pathway, as well as targeting pro-survival oncoproteins such as Bcl2, has shown efficacy as monotherapies for DLBCL (42). However, relapse as well as tumor dissemination to the bone marrow, kidney, liver, or the central nervous system can occur in advanced DLBCL and is associated with poor prognosis (42, 44, 51, 52). This underscores the urgent need for new therapeutics to treat DLBCL. Identifying novel drug targets for limiting DLBCL growth and dissemination could also facilitate the development of combination therapies with increased efficacy and reduced toxicity.

Tpm3.1/3.2 has been proposed as a target for cancer therapy. *Tpm3* expression is increased in many types of tumors relative to their normal cell counterparts (53, 54). Upon oncogenic transformation of fibroblasts, Tpm3.1/3.2 is maintained at high levels, whereas other Tpm isoforms are downregulated (30). Consistent with a role for Tpm3.1/3.2 in cancer progression, the proliferation, migration, invasion, expression of epithelial-to-mesenchymal transition markers, and *in vivo* tumor growth of esophageal squamous cell carcinoma cell lines are increased by *Tpm3* overexpression and decreased by *Tpm3* knockdown (55). Moreover, the Tpm3.1/3.2 inhibitor ATM-3507 (Anisina) (29, 30) inhibits the *in vitro* survival, growth, and motility of the B16-F1, C8161, WM164, S462, CMTRL-100, and ST88–14 tumor cell lines, and reduces the growth of human melanoma xenografts in immunodeficient mice (30, 31, 56).

ATM-3507 is a selective inhibitor of Tpm3.1/3.2 tropomyosin isoforms. Its parent compound, TR-100 (30, 57), was selected for its ability to bind a pocket in the C-termini of Tpm3.1 and Tpm3.2 that is encoded by exon 9d of the *Tpm3* gene. Exon 9d is not included in any of the other Tpm3 isoforms (28). ATM-3507 was developed by modifying the structure of TR-100 to enhance interactions with Tpm3.1/3.2-specific amino acid side chains and with the coiled-coil overlap junction between Tpm3.1/3.2 dimers, which is not shared with other Tpm isoforms (29). ATM-3507 intercalates between the actin filament and Tpm3.1/3.2 co-filament and alters the lateral movement of Tpm3.1/3.2 dimers across the actin filament. This inhibits the ability of Tpm3.1/3.2 to stabilize actin filaments, resulting in accelerated filament depolymerization (29). The ATM-3507-induced alteration in actin-Tpm3.1/3.2 co-filament interactions also disrupts the ability of Tpm3.1/3.2 to bridge actin filaments to myosin (25). Hence, ATM-3507 is a useful tool for revealing the actin- and actomyosin-dependent functions of Tpm3.1/3.2.

*Tpm3* is overexpressed in DLBCL relative to normal B cells (54), and a genome-wide CRISPR screen identified *Tpm3* as one of >150 genes that support the *in vitro* growth of DLBCL cell lines (58). However, the role of Tpm3.1/3.2 in regulating the survival, proliferation, and migration of malignant B cells has not been investigated. In this study, we show that ATM-3507 impairs BCR-induced actin remodeling and inhibits the *in vitro* growth and motility of DLBCL cells.

## 2 Materials and methods

### 2.1 Cells

The A20 IgG^+^ murine B-lymphoma cell line (ATCC #TIB-208; https://www.cellosaurus.org/CVCL_1940) and the B16-F1 murine melanoma cell line (ATCC #CRL-6323; https://www.cellosaurus.org/CVCL_0158) were obtained from ATCC (Manassas, VA, USA). The NU-DUL-1 (ATCC #CRL-2631; https://www.cellosaurus.org/CVCL_1877), Toledo (ATCC #CRL-2969; https://www.cellosaurus.org/CVCL_3611), and SU-DHL-8 (ATCC #CRL-2961; https://www.cellosaurus.org/CVCL_2207) human DLBCL cell lines were provided by Dr. Andrew Weng (BC Cancer Agency, Vancouver, Canada) and are available from ATCC (Manassas, VA, USA). The TMD8 human DLBCL cell line (https://www.cellosaurus.org/CVCL_A442) was provided by Dr. Neetu Gupta (Cleveland Clinic Lerner Research Institute, Cleveland, OH, USA). Murine primary B cells were isolated from the spleens of 10- to 13-week-old C57BL/6J mice (Jackson Laboratories, Bar Harbor, ME, USA #000664) in accordance with protocols approved by the University of British Columbia Animal Care Committee. Highly enriched B-cell populations were obtained using a negative selection B-cell isolation kit (Stemcell Technologies, Vancouver, BC, Canada #19854A), as described previously (59). B cell lines were cultured in RPMI-1640 supplemented with heat-inactivated fetal calf serum (FCS; 5% for A20 cells, 10% for all other cell lines), 50 µM 2-mercaptoethanol, 2 mM glutamine, and 1 mM pyruvate (culture medium). Upon isolation, primary B cells were cultured in culture medium containing 5 μg/mL *E. coli* 0111:B4 lipopolysaccharide (LPS; Sigma-Aldrich, St. Louis, MO, USA #L2630) for 6–18 h before being used for experiments. B16-F1 cells were cultured in DMEM with 10% FCS, 2 mM glutamine, and 1 mM pyruvate.

### 2.2 Cell spreading, immunofluorescence, and microscopy

Round glass coverslips (12 mm or 18 mm diameter) were coated with PBS containing 2.4 μg/cm^2^ goat anti-mouse IgG (for A20 cells; Jackson ImmunoResearch, West Grove, PA, USA #115-005-008), 2.4 μg/cm^2^ goat anti-mouse IgM (for primary B cells; Jackson ImmunoResearch, West Grove, PA, USA #115-005-020), or 0.625 μg/cm^2^ goat anti-mouse IgG plus 0.15 µg/cm^2^ ICAM-1 (Sino Biological Inc., Beijing, China #50440-M08H) for 30 min at 37°C. Afterwards, the coverslips were blocked for 30 min at room temperature with PBS containing 2% bovine serum albumin (BSA). Cells were resuspended to a 10^6^ cells/mL in PBS + 2% FCS (imaging medium), and 10^5^ cells (in 100 μL) was added to each coverslip. Where indicated, the cells were pre-treated with the Tpm3.1/3.2 inhibitor ATM-3507 (AdooQ Bioscience, Irvine, CA, USA #A2032), the myosin II inhibitor S-nitro-blebbistatin (snBB) (Cayman Chemicals, Ann Arbor, MI, USA #13891), or DMSO (solvent control) for 1 h at 37°C before adding the cells and the inhibitor-containing imaging medium to the coverslip. The cells were allowed to spread at 37°C for the indicated times before adding 100 µL of 8% paraformaldehyde to yield a final concentration of 4%. After fixing the cells for 15 min at room temperature, they were permeabilized with 0.2% Triton X-100 in PBS for 5 min. F-actin was stained with rhodamine-conjugated phalloidin (Thermo Fisher, Waltham, MA, USA #R415; 1:400 in PBS + 2% BSA; 30 min at room temperature). Cell areas and circularity indices were quantified from thresholded binary images using Fiji software version 2.14.0/1.54f (60). The outer edge of the peripheral F-actin defined the cell perimeter, and the cell area in the confocal plane closest to the coverslip was quantified. The circularity index is defined as 4π (area/perimeter²), and a value of 1.0 indicates a perfect circle. To determine the subcellular localization of Tpm3.1/3.2 relative to F-actin, cells were stained with a rabbit Ab to Tpm3 (Antibodies.com, Cambridge, UK #A13375; 1:200 in PBS + 2% BSA) for 1 h at room temperature, followed by staining with Alexa Fluor 488-conjugated goat anti-rabbit IgG (Invitrogen, Waltham, MA, USA #A-11008; 1:400 in PBS + 2% BSA) plus rhodamine-phalloidin (1:400) for 30 min at room temperature. Coverslips were mounted onto slides using ProLong Diamond anti-fade reagent (Thermo Fisher, Waltham, MA, USA #P36965). Images of cell-coverslip interfaces were captured using a spinning disk confocal microscope (Zeiss Axiovert 200M) with a 100× NA 1.4 oil-immersion objective lens. Manders’ co-localization coefficients were determined using the Coloc2 ImageJ plug-in. The percent of cells that formed prominent linear actin arc structures adjacent to the inner face of the peripheral actin ring was determined visually. Slides were viewed in random order, and only cells with clear and obvious actin arcs were scored as positive. Where indicated, stimulated emission depletion (STED) microscopy was performed as described previously (61) using a Leica TCS SP8 laser scanning STED system with a 592 nm depletion laser, a CX PL APO 100× NA 1.40 oil objective, and a Leica HyD high-sensitivity detector. Images were deconvoluted using Huygens software version 21.04 (Scientific Volume Imaging, Hilversum, The Netherlands).

### 2.3 Alamar Blue metabolic activity assay

The redox indicator Alamar Blue (62) was used to quantify metabolic activity. NU-DUL-1, TMD8, SU-DHL-8, or Toledo cells (2 × 10^4^ in 100 µL culture medium) were cultured in triplicate wells of a 96-well plate with ATM-3507 or equivalent volumes of DMSO. Alamar Blue HS reagent (10 µL per well; Thermo Fisher, Waltham, MA, USA #A50100) was added for the last 4 h of culture. Cell-mediated reduction of the Alamar Blue reagent was measured using a Varioskan LUX fluorescence microplate reader (Thermo Fisher, Waltham, MA, USA) with an excitation wavelength of 535 nm and an emission wavelength of 580 nm. For each cell line, the Alamar Blue fluorescence output was linear up to 1.6 x 10^5^ cells per well. Data were analyzed using Microsoft Excel.

### 2.4 Cell size and DNA content

To visualize cell size and nuclear size by confocal microscopy, DLBCL cells were cultured in 96-well plates (2 × 10^4^ per well) for the indicated times before being added to coverslips that had been coated with 10 µg/mL poly-L-lysine. After allowing the cells to attach for 30 min, they were fixed, permeabilized, and stained with rhodamine-phalloidin (as described in section 2.2) plus DAPI (1:10,000). To quantify cell size and DNA content by flow cytometry, DLBCL cells (2 × 10^5^ in 1 mL) were resuspended in culture medium with ATM-3507 or DMSO and added to wells of a 24-well plate. After 24 h, the cells were stained with 7-AAD (Sigma-Aldrich, St. Louis, MO, USA #129935; 1:1000) on ice and analyzed using a BD LSR II flow cytometer. Forward scatter (FSC), side scatter, and 7-AAD were used to gate on single live cells. The geometric mean of the FSC value was used as a measure of cell size. For quantifying DNA content, cells were pelleted by centrifugation and fixed with 70% ice-cold ethanol for 30 min on ice. The cells were then centrifuged at 800 x g for 10 min and resuspended in 1 mL PBS with 20 μg/mL RNase A (Invitrogen, Waltham, MA, USA #12091-021) for 15 min on ice before being stained with 10 µg/mL propidium iodide (PI; Invitrogen, Waltham, MA, USA #P1304) for 15 min on ice. PI fluorescence was quantified using a BD LSR II flow cytometer. Flow cytometry data were analyzed using FlowJo™ Software version 10.9.0 (BD Life Sciences, Franklin Lakes, NJ, USA).

### 2.5 Transwell migration assays and CXCR4 cell surface expression

NU-DUL-1 or Toledo DLBCL cells (5 × 10^5^) were pre-treated with ATM-3507 or the highest equivalent concentration of DMSO for 1 h at 37°C in 100 µL imaging medium. The cells were then added to a Transwell™ plate insert with 5-µm pores (Corning Life Sciences, Corning, NY, USA #3421). Imaging medium (600 µL) with the same concentration of ATM-3507 or DMSO plus 100 nM recombinant human CXCL12 (R&D Systems, Minneapolis, MN, USA #460-SD) was added to the bottom chamber. After allowing the cells to migrate for 4 h at 37°C, the cells in the bottom chamber were collected and counted for 120 s using a BD LSR II flow cytometer. The cells remaining in the top chamber were stained with 7-AAD, as described in the previous section, to assess their viability at the end of the assay. To quantify cell surface CXCR4 levels, cells were cultured with DMSO or ATM-3507 for the indicated times before being treated with human BD Fc block (BD Pharmingen, San Diego, CA, USA #564220; 0.5 μg/10^6^ cells) for 5 min, followed by staining with allophycocyanin (APC)-conjugated anti-human CD184 (CXCR4; BioLegend, San Diego, CA, USA #306510) for 30 min on ice. Cells were analyzed using a BD LSR II flow cytometer, and the data were analyzed using FlowJo™ software.

### 2.6 Two-dimensional motility

The wells of μ-Slide 8-well chambers (ibidi, Fitchburg, WI, USA #80826) were coated with 0.5 µg/cm^2^ bovine fibronectin (FN; Sigma-Aldrich, St. Louis, MO, USA #4759) for 1 h at 37°C. Toledo cells (3 × 10^4^) were resuspended in 300 µL imaging medium containing 100 nM CXCL12 plus either ATM-3507 or DMSO. The cells were then added to the FN-coated μ-Slide, which was placed in a 37°C Chamlide imaging chamber (Quorum Technologies, Puslinch, Ontario, Canada) with a 5% CO_2_ atmosphere. After 1 h, time-lapse images were acquired at 20X magnification every 30 s for 1 h using a Leica DM14000B microscope. Cell tracks were generated and analyzed using the Fiji TrackMate plug-in (version 2.14.0/1.54f; https://imagej.net/plugins/trackmate/). ICY software (https://icy.bioimageanalysis.org) was used to quantify the total distance and net displacement for individual cell tracks. Tracks that continued out of the field of view, tracks in which cells detached from the substrate, and tracks of dead cells were removed manually. Segmented tracks were joined manually. The ibidi Chemotaxis and Migration Tool (https://ibidi.com/chemotaxis-analysis/171-chemotaxis-and-migration-tool.html) was used to generate plots of cell tracks.

### 2.7 Statistical analysis

Prism GraphPad (version 10.4.0) software was used for statistical analyses. Ranked values in samples with large numbers of cells were compared using the Mann-Whitney U test. Outlier values were removed using Robust Regression and Outlier Removal (ROUT) in GraphPad Prism, with Q set to 1% (63). To compare values for matched sets of samples, e.g., median values from multiple experiments, two-tailed paired *t*-tests were used.

## 3 Results

### 3.1 Tpm3.1/3.2 is important for BCR-induced B cell spreading

**Figure 1:**
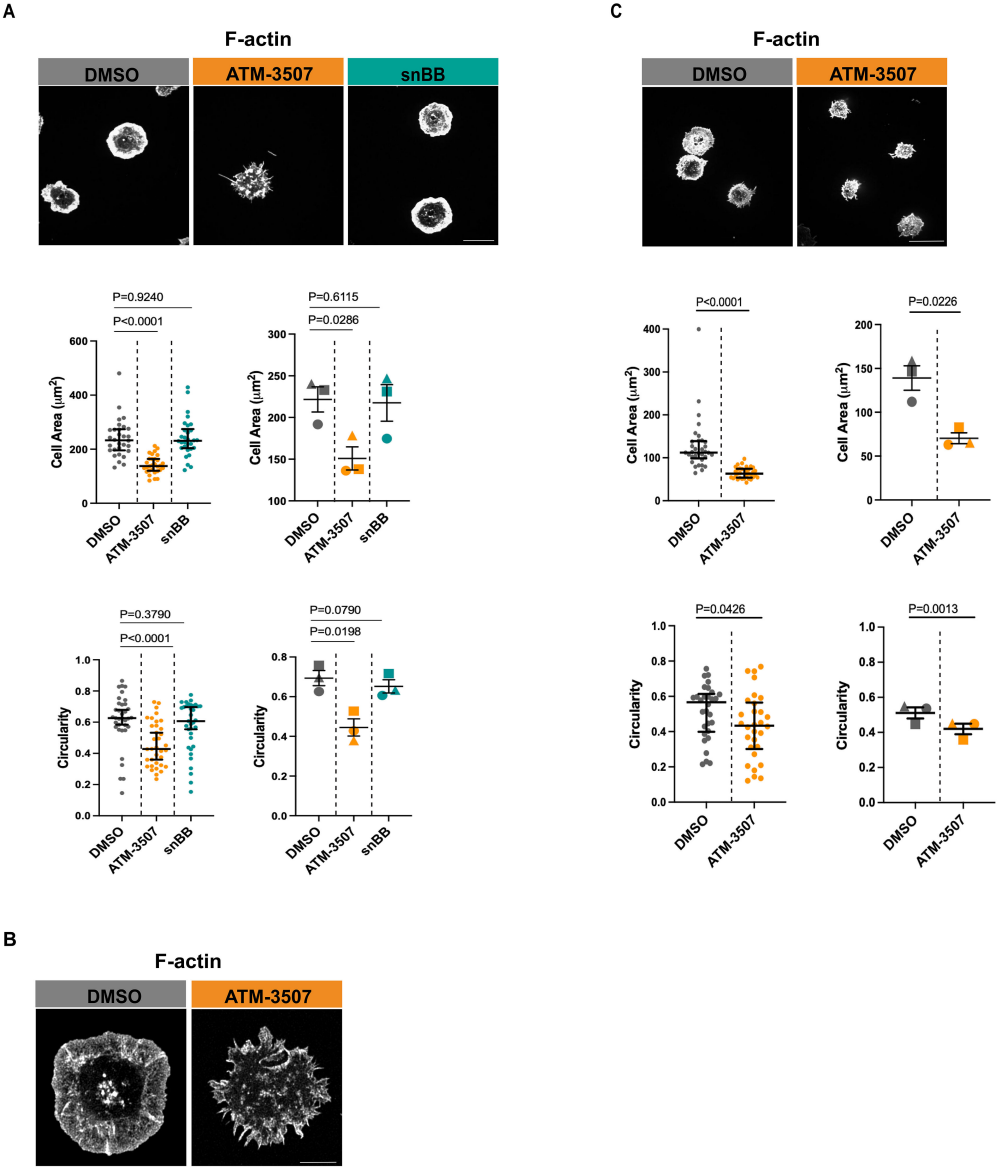
ATM-3507 treatment alters BCR-induced actin remodeling and impairs B cell spreading. **(A)** A20 cells were pre-treated with either 3 µM ATM-3507, an equivalent volume of DMSO (0.03% final concentration), or 50 µM snBB for 1 h at 37°C. The cells were added to coverslips coated with 2.4 μg/cm^2^ anti-IgG and allowed to spread for 30 min before being stained with rhodamine-phalloidin to visualize F-actin. For each condition, more than 105 cells from 3 independent experiments were imaged by confocal microscopy. Representative confocal microscopy images are shown (top panel; scale bar is 10 µm). Cell areas (middle panel) and cell circularity (lower panel) were quantified from the confocal microscopy images using ImageJ. The graphs on the left show one of three independent experiments (biological replicates) with similar results. Each dot represents one cell, and the medians and interquartile ranges are shown for >35 cells per condition. *p-values* were calculated using the Mann–Whitney U test. The graphs on the right show combined data from the 3 independent experiments. Each symbol is an individual experiment, and the data are presented as the mean ± SEM for the median values from the 3 experiments. *p-values* were calculated using two-tailed paired *t*-tests. **(B)** A20 cells were pre-treated with either 3 µM ATM-3507 or an equivalent volume of DMSO (0.03% final concentration) for 1 h at 37°C. The cells were added to coverslips coated with 2.4 μg/cm^2^ anti-IgG and allowed to spread for 30 min before being stained with rhodamine-phalloidin. For each condition, 15–20 cells were imaged by STED super-resolution microscopy. Representative images are shown (Scale bar is 5 µm). **(C)** LPS-activated primary murine B cells were pre-treated with either 3 µM ATM-3507 or 0.03% DMSO for 1 h at 37°C before being added to coverslips coated with 2.4 μg/cm^2^ anti-IgM for 30 min and then stained with rhodamine-phalloidin. For each condition, more than 105 cells from 3 independent experiments were imaged by confocal microscopy. Representative confocal microscopy images are shown (top panel; scale bar is 10 µm). Cell areas (middle panel) and cell circularity (lower panel) were quantified from the confocal microscopy images, and the data are presented as in panel **(A)**.

Upon contact with anti-Ig-coated surfaces, BCR signaling initiates actin remodeling that results in lamellipodia-driven radial cell spreading (61, 64). This response simulates the early phases of B-cell-APC interactions. The A20 murine B-lymphoma cell line, which has been referred to as a mouse model of DLBCL (65), has been widely used to identify proteins that mediate BCR-induced actin remodeling (13, 61, 64, 66–70). Because BCR-induced spreading has been extensively characterized in A20 cells, and defects in this process are readily detectable, we chose this cell line as a robust, sensitive, and well-defined system for our initial studies of the role of Tpm3.1/3.2 in regulating the actin cytoskeleton in B cells. To test if Tpm3.1/3.2 is involved in BCR-induced B cell spreading, we employed the pharmacological inhibitor ATM-3507 to selectively target these isoforms. We found that treating A20 cells (**Figure 1A**) with ATM-3507 significantly reduced their ability to spread on coverslips that had been coated with 2.4 µg/cm^2^ of anti-IgG Abs. The spreading response is driven by the formation of a peripheral F-actin ring, where Arp2/3 complex-nucleated branched actin polymerization exerts outward force on the plasma membrane (13). Hence, we asked if the reduced spreading in ATM-3507-treated cells was associated with impaired formation of this peripheral actin ring. Indeed, both confocal microscopy (**Figure 1A**) and STED super-resolution microscopy (**Figure 1B**) revealed that the thick, highly branched peripheral actin ring present in control A20 cells was virtually absent in the ATM-3507-treated A20 cells. Moreover, instead of a smooth lamellipodial leading edge around the entire periphery of the cells, ATM-3507-treated A20 cells exhibited multiple thin membrane protrusions (**Figures 1A, B**) and, therefore, had a significantly lower circularity index than DMSO-treated control cells (**Figure 1A**, lower panel). This altered peripheral actin organization could account for the impaired spreading that was observed when Tpm3.1/3.2 is inhibited by ATM-3507. We then extended this analysis to LPS-activated primary murine B cells. ATM-3507-treated primary B cells also exhibited impaired BCR-induced spreading when plated on immobilized anti-Ig (**Figure 1C**) and had a decreased circularity index compared to control cells (**Figure 1C**), indicative of altered actin dynamics and organization.

Because Tpm3.1/3.2 links actin filaments to myosin II, the impaired spreading in ATM-3507-treated cells could be due to a loss of actomyosin contractility. To test this, we treated A20 cells with the blebbistatin derivative snBB, which inhibits the ATPase activity of myosin II (71) and prevents actomyosin contractility. In contrast to ATM-3507, pre-treating A20 cells with 50 µM snBB for 1 h did not significantly reduce anti-Ig-induced cell spreading nor did it alter cell morphology, as indicated by the circularity index (**Figure 1A**). As a positive control for inhibition of myosin II by snBB, we showed that treating the B16-F1 melanoma cell line with snBB decreased the percent of cells with stress fibers from 66% to less than 3% (**Supplementary Figure 1**). Our finding that myosin II activity is not required for B cell spreading on immobilized anti-Ig is consistent with previous work showing that B cells lacking myosin II exhibit normal spreading on planar lipid bilayers to which Fab fragments of anti-Ig Abs had been tethered (72). Taken together, these findings suggest that the role of Tpm3.1/3.2 in promoting the radial spreading of B cells is not dependent on myosin or actomyosin contractility but instead reflects the ability of Tpm3.1/3.2 to stabilize actin filaments.

### 3.2 Tpm3 localizes to peripheral F-actin structures and supports the formation of actomyosin arcs

**Figure 2:**
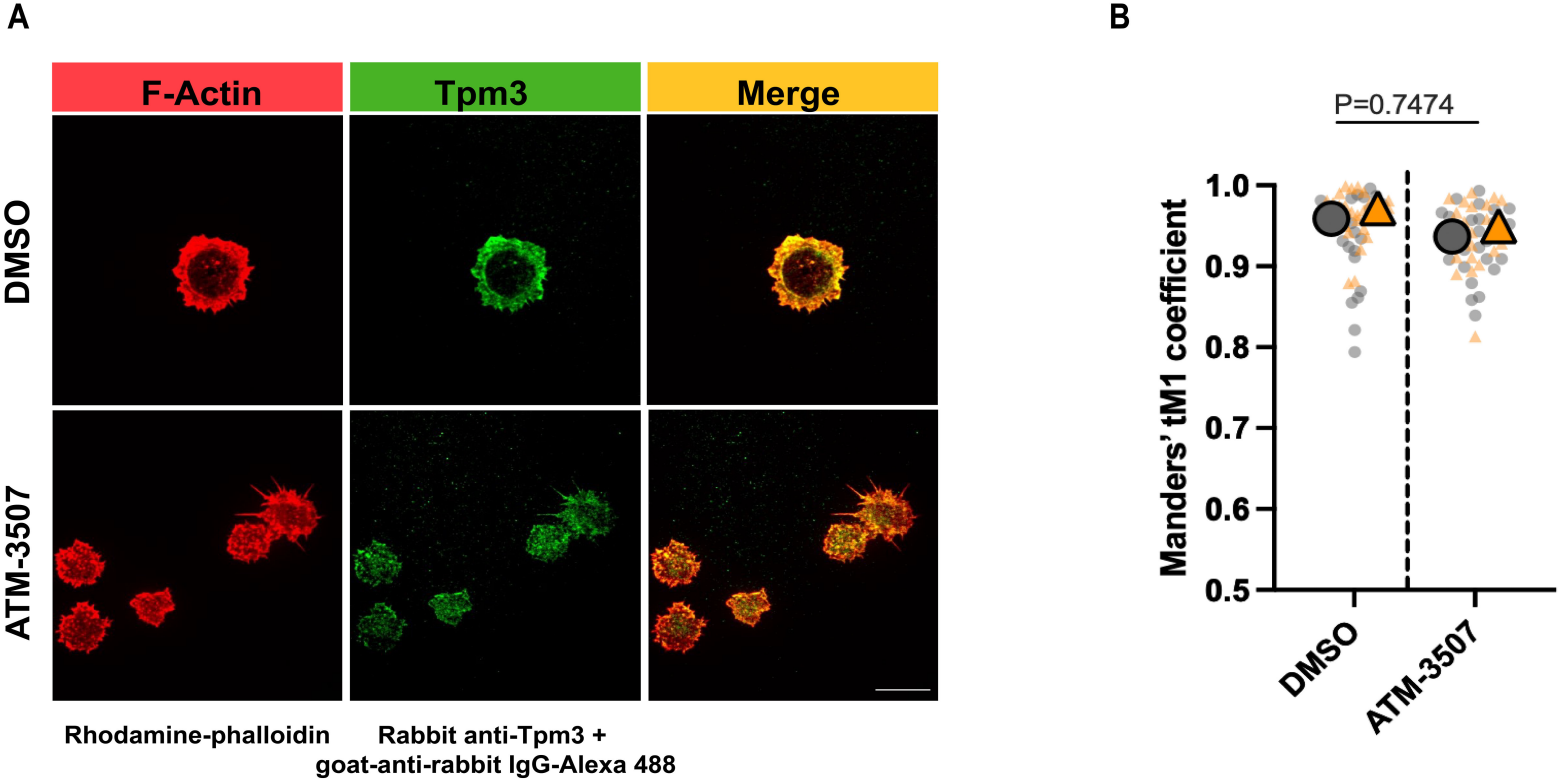
Tpm3 co-localizes with the peripheral F-actin ring during B cell spreading. A20 cells were pre-treated for 1 h with 0.03% DMSO or 3 µM ATM-3507 and then allowed to spread for 30 min on coverslips that had been coated with 2.4 µg/cm^2^ anti-IgG. Cells were stained for F-actin (rhodamine-phalloidin) and Tpm3 and then imaged by confocal microscopy. **(A)** Representative images from one of 3 independent experiments. Scale bar: 10 µm. **(B)** The Manders’ coefficient represents the fraction of Tpm3 staining that co-localized with F-actin staining. The super-plot displays combined data for >25 cells per condition from two independent experiments. Each dot is one cell, and the different colors represent the two experiments. The large symbols represent the median values for each experiment. *p-values* for DMSO versus ATM-3507-treated cells were calculated using the Mann–Whitney U test.

Because ATM-3507 treatment disrupted the formation of the peripheral actin ring that drives B cell spreading, we asked whether Tpm3.1/3.2 normally localizes to this structure. Immunostaining with a polyclonal Ab that recognizes multiple Tpm3 isoforms showed that Tpm3 strongly co-localized with the peripheral actin ring in A20 cells that had spread on coverslips coated with 2.4 µg/cm^2^ anti-IgG (**Figure 2A**). In contrast, when A20 cells were treated with ATM-3507, the F-actin organization was altered, and Tpm3 was located primarily in punctae throughout the cell body and in the filopodia-like protrusions (**Figure 2A**). Nevertheless, a high degree of Tpm3 co-localization with F-actin was still evident in the ATM-3507-treated cells. The Manders’ coefficients for the co-localization of Tpm3 with F-actin were similar in the ATM-3507- and DMSO-treated cells (**Figure 2B**). This is consistent with the idea that Tpm3 polymers are assembled onto actin filaments and that the organization of actin filaments within the cell determines the localization of Tpm3 polymers (17). Moreover, because ATM-3507 abrogates the ability of Tpm3.1/3.2 to protect actin filaments from cofilin-mediated severing, but does not dissociate Tpm3.1/3.2 from F-actin (57), the apparent co-localization of F-actin and Tpm3 in the punctae observed in ATM-3507-treated A20 cells could be severed fragments of actin-Tpm3 co-filaments.

**Figure 3:**
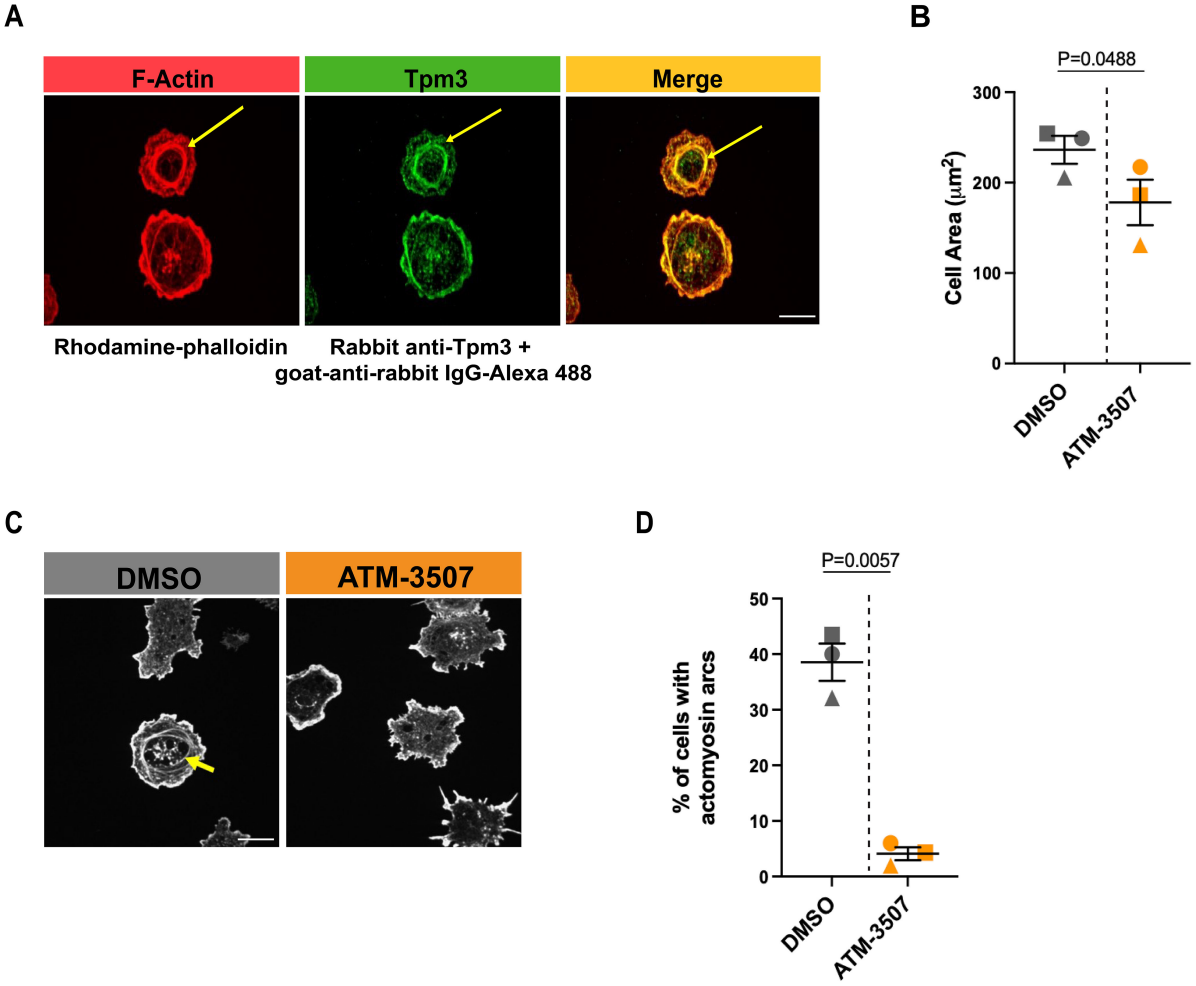
Tpm3 co-localizes with actin arcs, and Tpm3.1/3.2 function is important for their formation. **(A)** A20 cells were allowed to spread for 30 min on coverslips that had been coated with 0.625 µg/cm^2^ anti-IgG plus 0.15 µg/cm^2^ ICAM-1. Cells were stained for F-actin (rhodamine-phalloidin) and Tpm3 and then imaged by confocal microscopy. Representative images from one of 3 independent experiments are shown, in which yellow arrows indicate an actin arc. Scale bar: 10 µm. **(B-D)** A20 cells were pre-treated for 1 h with 0.03% DMSO or 3 µM ATM-3507 and then allowed to spread for 30 min on coverslips coated with 0.625 µg/cm^2^ anti-IgG plus 0.15 µg/cm^2^ ICAM-1. F-actin staining was used to define the cell periphery, quantify cell areas, and visualize actin arcs. In **(B)**, each symbol represents the median value from one of 3 independent experiments in which n >30 cells per condition were analyzed. The mean ± SEM for the median values from the 3 experiments is shown. Representative images of DMSO- and ATM-3507-treated cells are shown in **(C)**, with an actin arc indicated by a yellow arrow. The percent of DMSO- and ATM-3507-treated cells in which actin arcs were clearly visible is shown in **(D)**. Each symbol represents the median value from one of 3 independent experiments in which n >30 cells per condition were analyzed. The mean ± SEM for the median values from the 3 experiments is shown. *p-values* in panels **(B)** and **(D)** were calculated using two-tailed paired *t*-tests.

High densities of immobilized anti-Ig Abs cause strong BCR signaling that promotes B-cell spreading. However, when B cells attach to surfaces with lower anti-Ig densities that are insufficient to induce cell spreading, the concomitant binding of the LFA-1 integrin to the ICAM-1 adhesion molecule can enable cell spreading (61, 68). Specifically, A20 cells spread when plated on coverslips coated with 2.4 µg/cm^2^ anti-IgG Abs, do not spread on coverslips coated with 0.625 µg/cm^2^ anti-IgG alone, but exhibit robust spreading on coverslips coated with 0.625 µg/cm^2^ anti-IgG plus ICAM-1 (61). This reflects synergy between low-level BCR signaling and LFA-1 signaling, as neither low-density anti-Ig alone nor ICAM-1 alone can stimulate B-cell spreading (61, 68). Moreover, the combination of suboptimal BCR signaling and LFA-1 signaling uniquely leads to the formation of actomyosin arcs at the inner face of the peripheral actin ring that forms as the cells spread (61, 68). At the B cell-APC immune synapse, these contractile actomyosin arcs sweep BCR microclusters toward the center of the cell-cell contact site, leading to the formation of a central supramolecular activation complex (cSMAC) (61, 68). We found that Tpm3 was highly enriched at these actin arcs when A20 cells spread on coverslips coated with ICAM-1 plus 0.625 µg/cm^2^ anti-IgG (**Figure 3A**), an anti-IgG density that does not support cell spreading by itself (61). To determine if targeting Tpm3.1/3.2 with ATM-3507 impacted the formation of these actin arcs, we visually assessed confocal microscopy images and quantified the percent of cells that formed prominent linear actin structures adjacent to the inner face of the peripheral actin ring, as done previously by ourselves and others (61, 68). ATM-3507 treatment reduced the extent of cell spreading when A20 cells were plated on ICAM-1 plus 0.625 µg/cm^2^ anti-IgG (**Figure 3B**). In addition, treating A20 cells with ATM-3507 almost completely abolished their ability to form actin arcs when they spread on ICAM-1 plus suboptimal anti-IgG (**Figures 3C, D**). Thus, Tpm3.1/3.2 plays an important role in the actin remodeling that drives B-cell spreading and is essential for the formation of actomyosin arcs at the inner face of the peripheral actin ring.

### 3.3 ATM-3507 inhibits DLBCL growth and cell division

**Figure 4:**
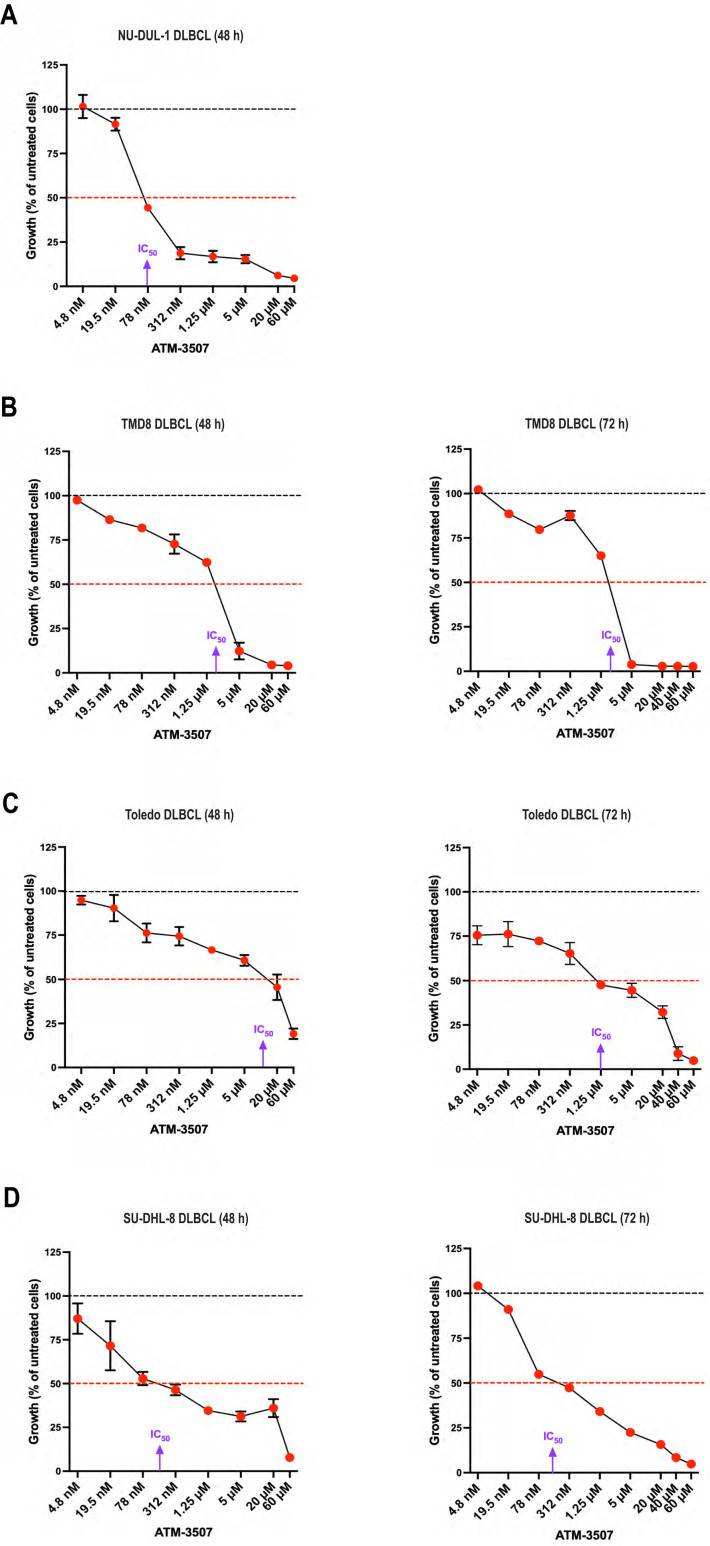
ATM-3507 inhibits the growth of DLBCL cell lines. NU-DUL-1 **(A)**, TMD8 **(B)**, Toledo **(C)**, or SU-DHL-8 DLBCL cells **(D)** were cultured for 48 h or 72 h with the indicated concentrations of ATM-3507. The Alamar Blue assay was used to measure metabolic activity. In each experiment, each condition was analyzed in triplicate wells (technical replicates), and the mean value was used for data analysis. The data are expressed as a percent of the metabolic activity for cells cultured in the absence of ATM-3507 (control; dotted black lines). Each symbol is the average ± range or the mean ± SEM from 2 or 3 independent experiments. Where no error bars are shown, they were smaller than the symbol. The dotted red lines indicate 50% inhibition. Approximate IC_50_ values are shown. DMSO concentrations equivalent to those for the highest ATM-3507 concentrations had no effect on metabolic activity.

Because Tpm3.1/3.2 has been implicated in cancer progression and the *Tpm3* gene is overexpressed in many cancers, including DLBCL (54), we hypothesized that Tpm3.1/3.2 could be a target for treating DLBCL. First, we used the Alamar Blue metabolic activity assay to investigate whether the Tpm3.1/3.2 inhibitor ATM-3507 could inhibit the growth of DLBCL cell lines. We found that ATM-3507 significantly reduced the growth of the NU-DUL-1, TMD8, Toledo, and SU-DHL-8 DLBCL cell lines (**Figure 4**). TMD8 is an ABC-DLBCL cell line that is dependent on chronic active BCR signaling, whereas SU-DHL-8 is a GCB-DLBCL cell line. NU-DUL-1 and Toledo are sometimes classified as ABC-DLBCL and GCB-DLBCL cell lines, respectively, but may be more similar to the unclassified group of DLBCLs based on their expression of early B-cell markers (73). The NU-DUL-1 DLBCL cell line was highly sensitive to growth inhibition by ATM-3507, with an IC_50_ value <100 nM and an IC_80_ value of ~300 nM in a 48-hour growth assay (**Figure 4A**). By 72 h, the majority of NU-DUL-1 cells treated with ATM-3507 concentrations of 78 nM or higher were dead. The TMD8 ABC-DLBCL cell line was less sensitive to ATM-3507. Although the IC_50_ values at both 48 h and 72 h were ~3 µM, ATM-3507 caused nearly complete growth inhibition at 5 µM (**Figure 4B**). Toledo cells were more resistant to the growth inhibitory effects of ATM-3507 than either NU-DUL-1 or TMD8 cells (**Figure 4C**). However, extending the treatment time resulted in the IC_50_ value decreasing from ~10 µM ATM-3507 at 48 h to 1.25 µM at 72 h. Finally, at both 48 h and 72 h, ATM-3507 inhibited the growth of SU-DHL-8 GCB-DLBCL cells by 50% at a concentration of ~200 nM (**Figure 4D**), with higher ATM-3507 concentrations causing greater growth arrest at 72 h than at 48 h (**Figure 4D**). Although each DLBCL cell line exhibited a distinct dose-response pattern for ATM-3507-induced growth arrest, 50% growth inhibition after 48–72 h was achieved with nanomolar to low-micromolar concentrations of the drug, indicating that ATM-3507 can inhibit the growth of cell lines that represent different DLBCL subtypes.

**Figure 5:**
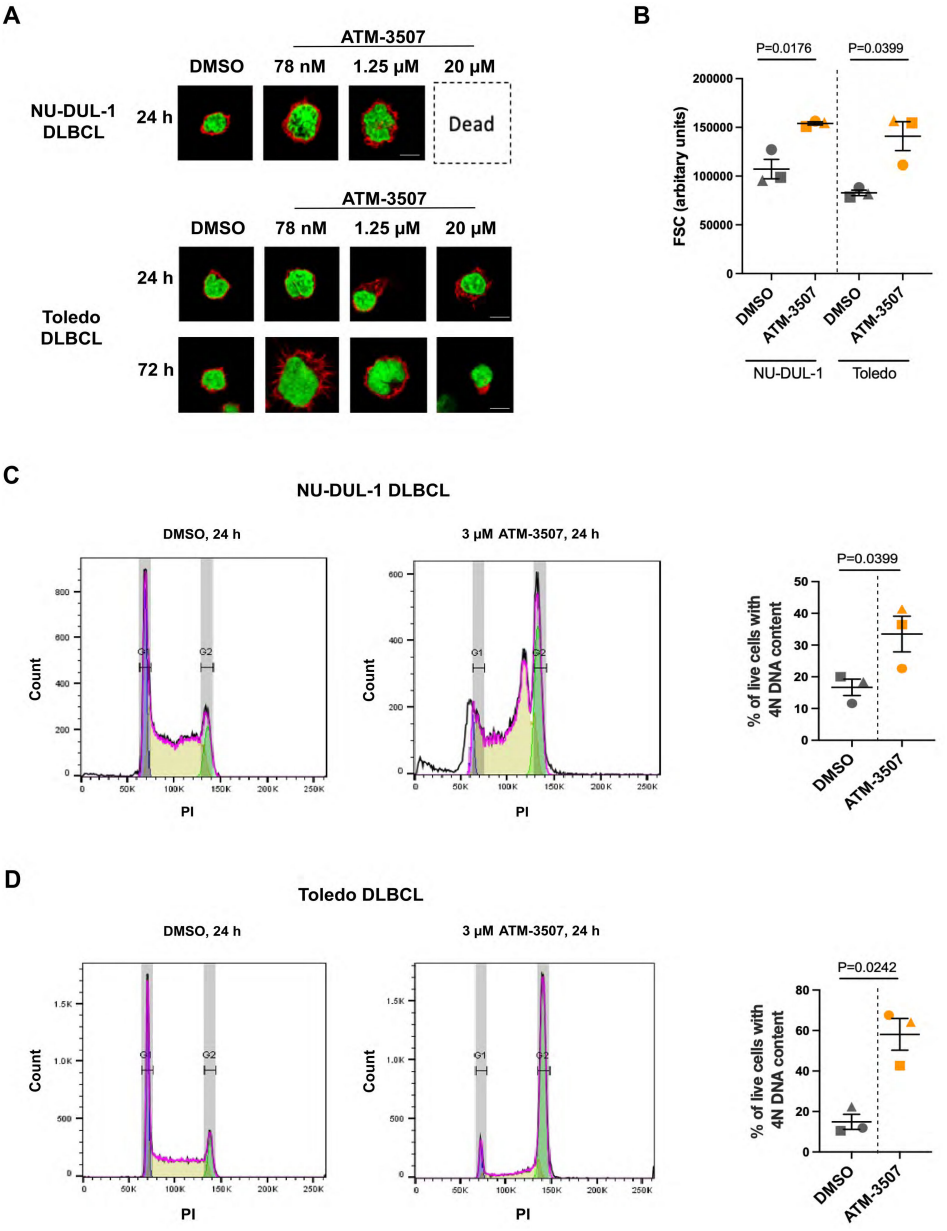
ATM-3507 causes G2/M cell cycle arrest in DLBCL cells. **(A)** NU-DUL-1 or Toledo cells were cultured with 0.03% DMSO or the indicated concentrations of ATM-3507 for 24 h or 72 h before being stained with rhodamine-phalloidin and DAPI. Cells were imaged by confocal microscopy. Representative images are shown. **(B)** NU-DUL-1 or Toledo cells were cultured for 24 h with 3 µM ATM-3507 or 0.03% DMSO before being analyzed by flow cytometry. Single live cells were identified by FSC, side scatter, and 7-AAD staining. Geometric means of the FSC values from 3 independent experiments are shown along with the mean ± SEM. **(C, D)** Propidium iodide (PI) staining was used to quantify the DNA content of live NU-DUL-1 **(C)** or Toledo **(D)** cells. Representative histograms showing peaks corresponding to cells with 2N or 4N DNA content. The graphs show the percent of live cells with 4N DNA content. Each symbol is an independent experiment, and the data are presented as the mean ± SEM from 3 experiments. *p-values* were calculated using two-tailed paired *t*-tests.

In addition, imaging DLBCL cells that had been co-stained with rhodamine-phalloidin (to visualize F-actin at the cell periphery) and DAPI showed that treating DLBCL cells with ATM-3507 concentrations as low as 78 nM or 1.25 µM, depending on the cell line, resulted in increased cell size as well as enlargement of the nucleus (**Figure 5A**, **Supplementary Figure 2**). These responses to ATM-3507 were observed for NU-DUL-1 and Toledo DLBCL cells (**Figure 5A**), as well as the SU-DHL-8 and TMD8 DLBCL cell lines (**Supplementary Figure 2**), after 24 h and were more evident by 72 h. To quantify the increase in cell size, we carried out flow cytometry, using the forward scatter (FSC) geometric mean as a measure of cell size. This showed that treating NU-DUL-1 or Toledo DLBCL cells with 3 µM ATM-3507 for 24 h resulted in a significant increase in FSC (**Figure 5B**). Because actin remodeling is essential for cell division (74), we hypothesized that the ATM-3507-induced increase in cell size and nuclear enlargement reflected cells that were arrested in the G2/M phase of the cell cycle and unable to divide. Staining cells with propidium iodide (PI) revealed that a 24-hour treatment with ATM-3507 resulted in a significant increase in the percentage of live NU-DUL-1 and Toledo cells with a 4N DNA content (**Figures 5C, D**), indicative of G2/M cell cycle arrest. This may be due to impaired cytokinesis when Tpm3.1/3.2 function is inhibited by ATM-3507, as has been observed in ATM-3507-treated ovarian cancer cells (75). The ATM-3507-treated DLBCL cells that arrest in G2/M phase subsequently die, as indicated by the loss of metabolic activity at 48 h and 72 h (**Figure 4**).

### 3.4 ATM-3507 inhibits DLBCL cell migration toward CXCL12 and motility on fibronectin

**Figure 6:**
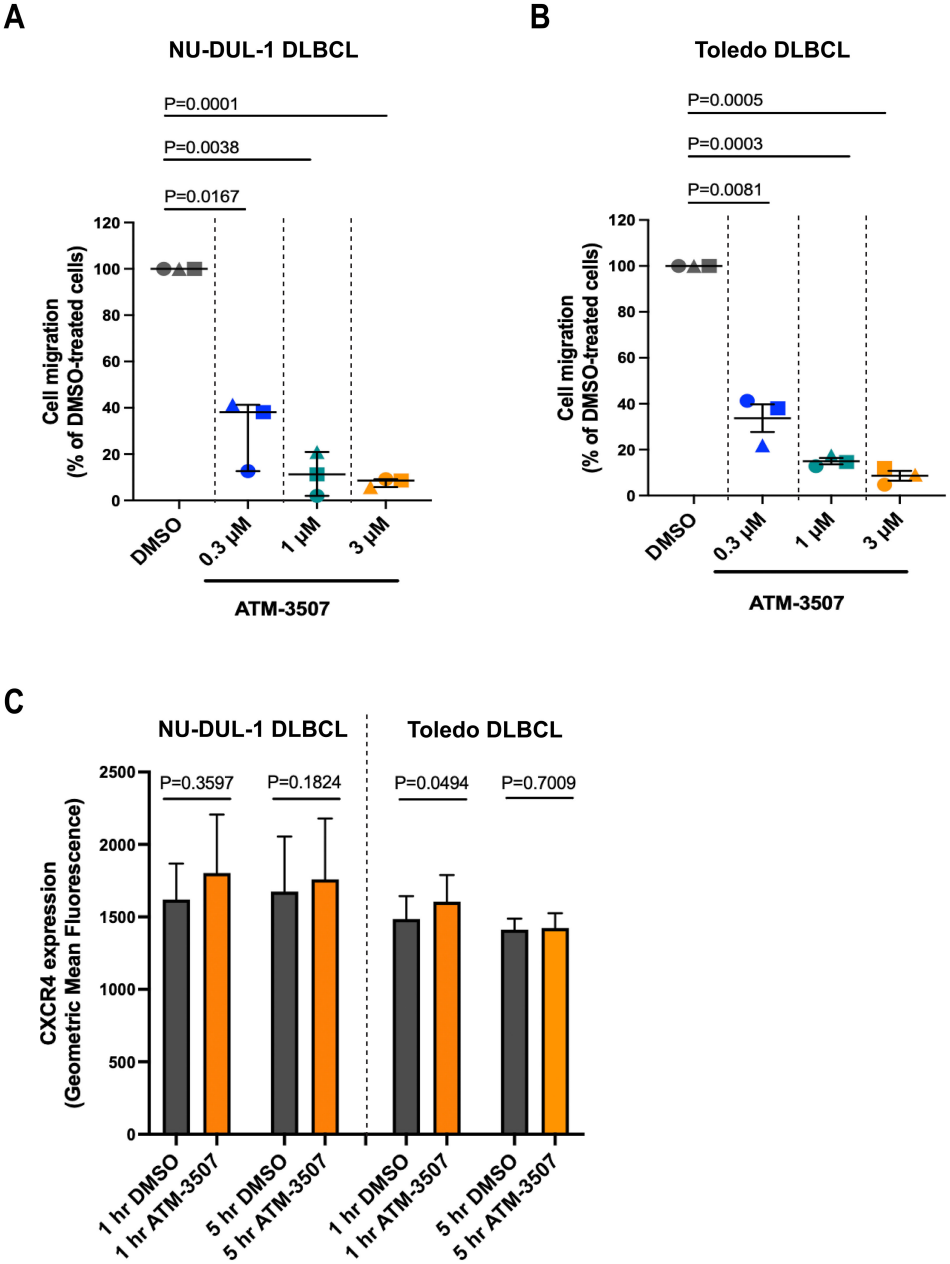
ATM-3507 inhibits the migration of DLBCL cells toward CXCL12. **(A, B)** NU-DUL-1 or Toledo cells were pre-treated with ATM-3507 or 0.03% DMSO (equivalent to the highest ATM-3507 concentration) for 1 h before being added to the upper chamber of a Transwell. The lower chamber contained 100 nM CXCL12 plus the same concentration of ATM-3507 or DMSO as in the upper chamber. After 4 h, the number of cells that had migrated into the lower chamber was determined using flow cytometry. The data are expressed as a percent of the number of DMSO-treated cells that migrated into the bottom chamber. The 100% values (percent of DMSO-treated cells that migrated into the lower chamber) in individual experiments ranged from 15-30% for NU-DUL-1 cells and from 50-70% for Toledo cells. Each symbol is an independent experiment. Means ± SEM are shown for 3 independent experiments. **(C)** NU-DUL-1 or Toledo cells were treated with 3 µM ATM-3507 or 0.03% DMSO for 1 h or 5 h before quantifying cell surface levels of CXCR4 by flow cytometry. Means ± SEM are shown for 3 independent experiments. *p-values* were calculated using two-tailed paired *t*-tests.

DLBCLs originate in lymph nodes but can subsequently disseminate to distant sites, a manifestation that is associated with disease progression and poor prognosis. Chemokines such as CXCL12 and CXCL13 support the trafficking of malignant B cells to other organs (76, 77) and activate the integrins that enable DLBCL cells to exit the vasculature and migrate within tissues (14). Because actin dynamics drive cell motility (78, 79), we hypothesized that inhibition of Tpm3.1/3.2 by ATM-3507 would impair both the chemotaxis and integrin-dependent motility of DLBCL cells. To assess chemotaxis, we used Transwell migration assays to quantify the ability of NU-DUL-1 and Toledo DLBCL cell lines to migrate toward the chemokine CXCL12. These cell lines exhibited robust responses in this assay, with 15-30% of NU-DUL-1 cells and 50-70% of Toledo cells migrating into the CXCL12-containing lower chamber of the Transwell. Importantly, we found that the ability of both Toledo cells and NU-DUL-1 DLBCL cells to migrate toward CXCL12 was significantly reduced by 0.3 µM ATM-3057 and inhibited by >90% by 3 µM ATM-3507 (**Figures 6A,B**). This was not due to cytotoxic effects of the drug, as the viability of the unmigrated cells in the top chamber at the end of the assay was >95% under all conditions. Moreover, the inhibition of CXCL12-induced migration by ATM-3507 was not due to decreased levels of cell surface CXCR4, the receptor for CXCL12 (**Figure 6C**).

**Figure 7:**
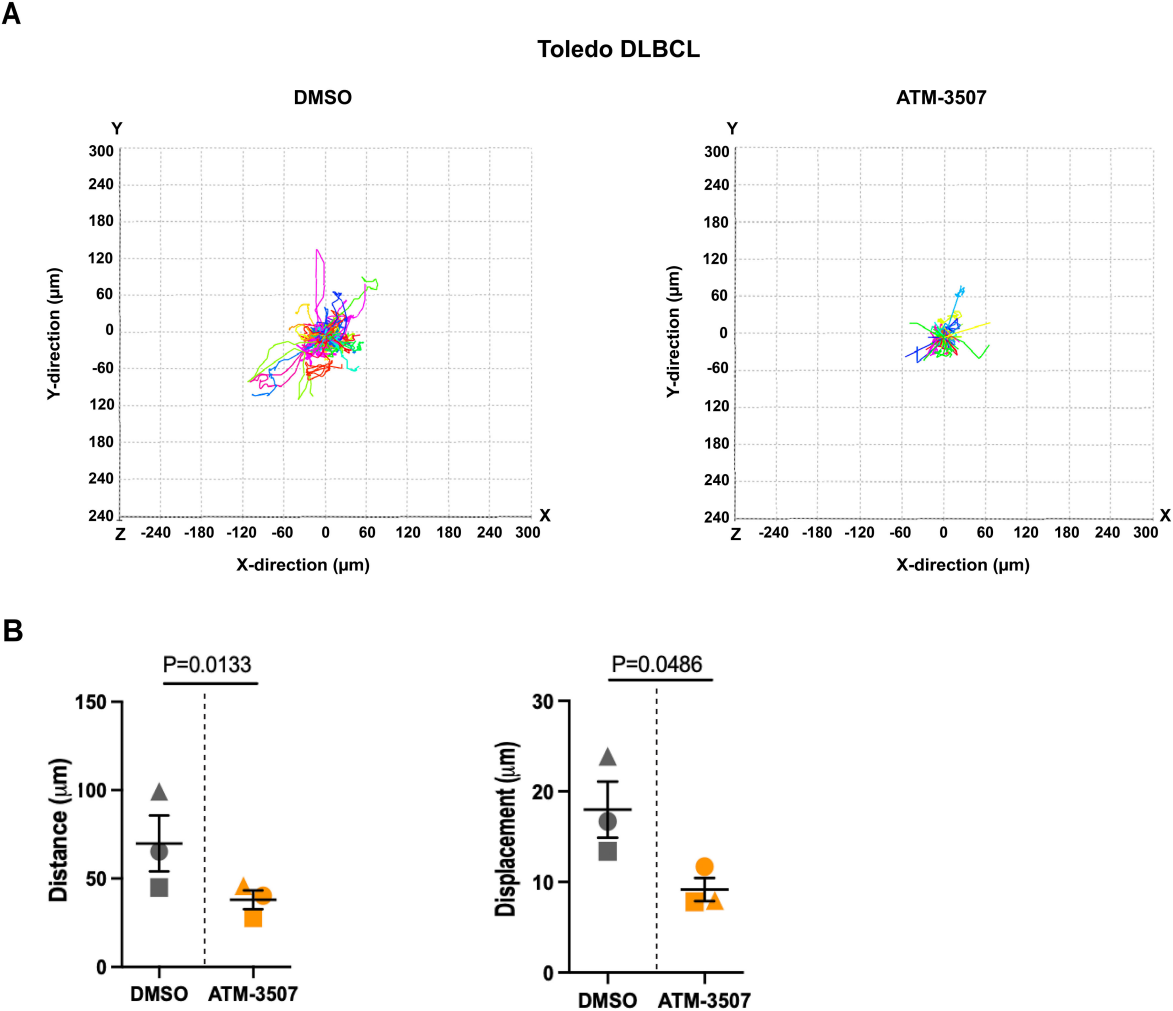
ATM-3507 inhibits the 2D motility of DLBCL cells on FN. Toledo DLBCL cells were added to FN-coated coverslips in the presence of 100 nM CXCL12 and either 10 µM ATM-3507 or 0.1% DMSO. After a 1 h pre-treatment period, time-lapse images were acquired every 30 s for 1 h. Cell tracks were generated from the time-lapse videos. Representative videos of DMSO-treated cells (**Supplementary Video 1**) and ATM-3507-treated cells (**Supplementary Video 2**) are in the **Supplementary Material**. **(A)** Individual cell tracks from a representative experiment. In each experiment, 40–100 cell tracks were analyzed per condition. **(B)** Compiled data from 3 independent experiments. Each symbol represents the median value from an individual experiment. Means ± SEM are shown. *p-values* were calculated using two-tailed paired *t*-tests.

To model the integrin-dependent movement of DLBCL cells across the surface of vascular endothelial cells or on extracellular matrices within tissues, we performed time-lapse imaging of DLBCL cells that were plated on FN-coated coverslips in the presence of CXCL12. FN is a ligand for the VLA-4 (α_4_β_1_) integrin on B cells. Many of the Toledo DLBCL cells extended lamellipodial protrusions, assumed a polarized “motile” morphology, and exhibited substantial net displacement over the one-hour observation period (**Supplementary Video 1**). ATM-3507 treatment inhibited the 2D motility of Toledo cells on FN (**Figure 7A**, **Supplementary Videos 1**, **2**), resulting in significant reductions in the median values for total track length and net displacement between the start and end of the track (**Figure 7B**). Although only a small fraction of NU-DUL-1 cells exhibited significant net displacement in these 2D motility assays, >90% of the cells (as determined visually; n = 169 cells from 5 independent experiments) transiently generated lamellipodial leading-edge membrane protrusions and a polarized morphology over the one-hour observation period (**Supplementary Video 3**). The cells appeared to be stuck in place, and distinct trailing-edge uropods that were unable to detach were observed (**Supplementary Video 3**). Strikingly, as shown in **Supplementary Video 4**, only 8% of the ATM-3507-treated NU-DUL-1 cells (n = 108 cells from 4 independent experiments) ever developed a polarized morphology over the one-hour observation period. This finding is consistent with the idea that ATM-3507-mediated inhibition of Tpm3.1/3.2 impairs the actin dynamics that generate leading-edge membrane protrusions. Taken together, **Figures 6**, **7** show that Tpm3.1/3.2 function is important for DLBCL cells to migrate toward chemokines, assume a polarized motile morphology, and undergo integrin-mediated cell motility.

## 4 Discussion

**Figure 8:**
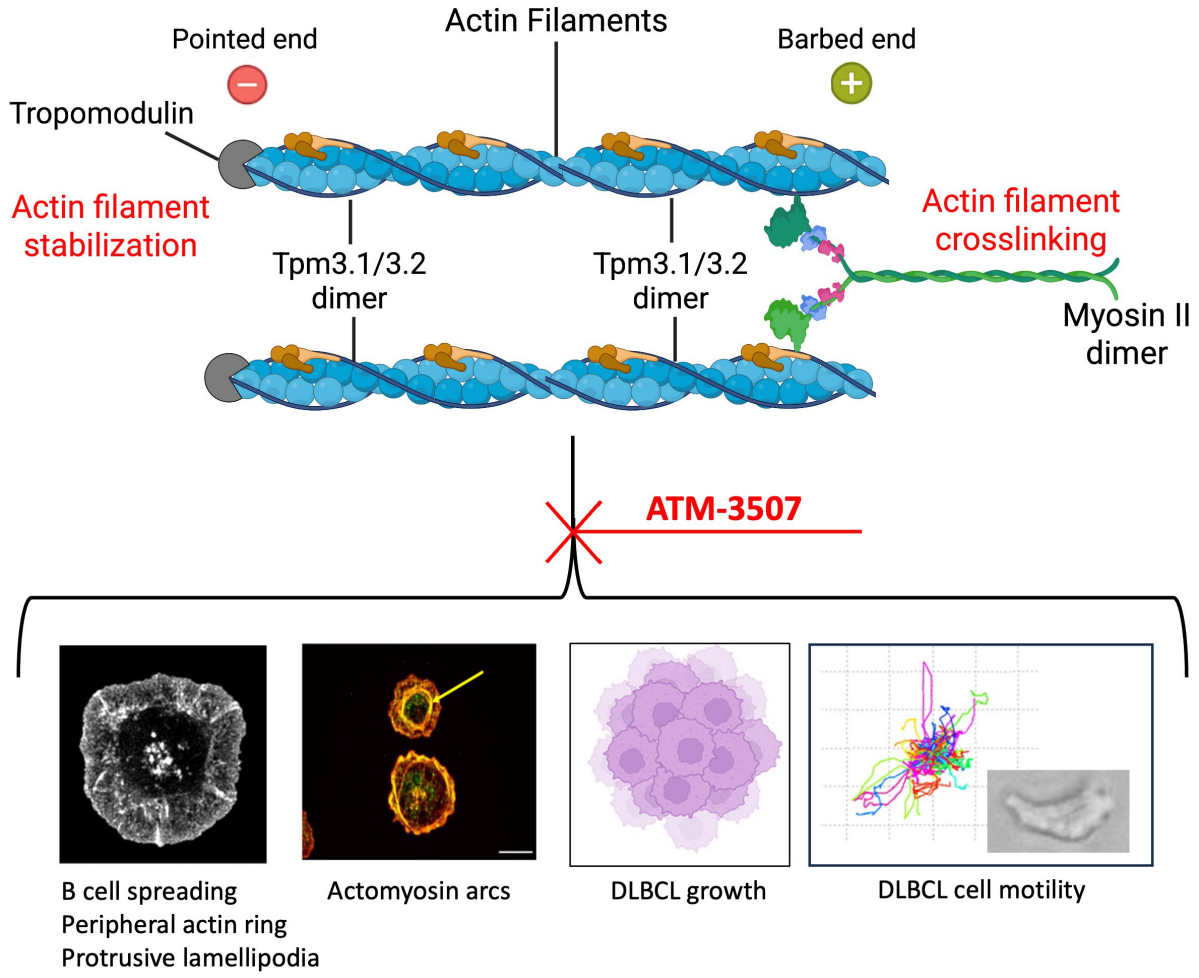
Tpm3.1/3.2 regulates actin organization and actin-dependent processes in B cells. Tpm3.1/3.2 dimers associate with actin filaments, form homopolymers along the filament, stabilize the actin filament, and recruit myosin II. Myosin II dimers can crosslink actin filaments and mediate actomyosin contractility. Inhibition of Tpm3.1/3.2 by ATM-3507 impairs multiple actin-dependent processes in B cells, including the assembly of a peripheral actin ring that drives BCR-induced cell spreading via the formation of protrusive lamellipodia, and the formation of actomyosin arcs (indicated by the yellow arrow) at the inner face of the peripheral actin ring. ATM-3507 also inhibits the growth of DLBCL cells as well as their CXCL12-dependent migration and motility. Created with BioRender.com.

In this study, we used ATM-3507 to probe the roles of Tpm3.1/3.2 in B-cell actin dynamics and to test the potential of ATM-3507 to inhibit the growth and dissemination of DLBCLs. We show that ATM-3507 impaired BCR-induced actin remodeling and cell spreading, as well as the growth, chemokine-induced migration, and integrin-mediated motility of DLBCL cells (**Figure 8**).

Our results suggest that Tpm3.1/3.2 is critical for B-cell spreading in response to BCR engagement. When spreading on immobilized anti-Ig, BCR signaling induces the assembly of a peripheral network of branched actin that exerts outward forces and forms broad lamellipodia (13, 61, 69). We found that Tpm3 was highly enriched in this peripheral actin ring, suggesting that it may function at that site to support lamellipodial protrusion. Indeed, when ATM-3507 was used to inhibit Tpm3.1/3.2, A20 cells did not generate this thick peripheral ring of branched actin or lamellipodia. Instead, they formed multiple thin, irregular actin-rich protrusions and exhibited a significant reduction in cell spreading compared to control cells. This suggests that the interaction of Tpm3.1/3.2 with F-actin contributes to the establishment or maintenance of the peripheral F-actin ring. In addition to cell spreading, the formation of protrusive leading-edge lamellipodia drives cell motility. Accordingly, we found that inhibiting Tpm3.1/3.2 function with ATM-3507 reduced leading-edge membrane protrusion and the 2D motility of DLBCL cells on FN substrates as well as their chemotaxis toward CXCL12. A role for Tpm3.1 in promoting leading-edge protrusion is supported by experiments showing that overexpressing Tpm3.1 in primary neurons results in enlarged neuronal growth cones (36).

Tpm3.1/3.2 could support the formation of protrusive actin networks in multiple ways. When Tpm3.1/3.2 forms co-filaments with actin, it decreases the rate of spontaneous actin filament depolymerization (23). Tpm3.1 also recruits tropomodulin (32), which prevents depolymerization at the pointed end of the actin filament and shields F-actin from the actin-severing protein cofilin (23). Additionally, Tpm3.1/3.2 recruits myosin II dimers that can crosslink actin filaments. Actin-crosslinking and actin-bundling proteins such as myosin II, α-actinin, and L-plastin stabilize actin networks and enable them to exert forces (80, 81) that generate lamellipodial protrusions and drive cell spreading and motility (82). ATM-3507 prevents Tpm3.1/3.2 from recruiting myosin dimers to actin (25), thereby ablating its actin-crosslinking function. This could reduce the stability of lamellipodia, causing them to collapse and retract more rapidly. Although the actin-bundling protein L-plastin increases the stability of lamellipodia in lymphocytes (83) and is required for chemokine-induced B-cell migration (84), our findings indicate that Tpm3.1/3.2 has unique non-redundant roles in B-cell spreading and motility.

The role of Tpm3.1/3.2 in promoting the formation of actomyosin arcs further underscores its importance in actin remodeling in B cells. Actomyosin arcs form at the inner face of the peripheral actin ring when B cells spread on surfaces displaying integrin ligands plus sub-optimal densities of anti-Ig that are insufficient to induce cell spreading (61, 68). The formation of these actomyosin arcs is a unique consequence of signaling by the LFA-1 integrin on B cells (68). Their myosin-based contractile function promotes the centripetal movement of BCR microclusters, and contributes to cSMAC formation when B cells form an immune synapse with APCs or membranes in which antigens are mobile (68). We found that Tpm3 co-localizes with these actin arcs when A20 cells undergo spreading on surfaces coated with ICAM-1 and a low density of anti-Ig. Importantly, treating A20 cells with ATM-3507 prevented the formation of these actin arc structures. When B cells undergo radial spreading on rigid surfaces, actomyosin arcs originate from linear, unbranched actin filaments that traverse the peripheral branched actin network and then extend along the inner face of the peripheral actin ring (68). The subsequent recruitment of myosin IIA dimers crosslinks additional linear actin filaments to create concentric rings that resemble stress fibers. Tpm3.1/3.2 preferentially decorates linear actin filaments assembled by formin proteins (17). Hence, during B-cell spreading, Tpm3.1/3.2 may play a key role in recruiting myosin II to formin-polymerized actin filaments at the inner face of the peripheral branched actin network, thereby establishing contractile actomyosin arcs. The Tpm3.1/3.2 inhibitor causes myosin II to dissociate from actin stress fibers in U2OS cells (25). Thus, the absence of actin arcs in ATM-3507-treated A20 cells could be a consequence of the destabilization of actin filaments within the peripheral ring of branched actin, impaired formation or decreased stability of formin-assembled linear actin filaments that comprise the actin arcs, or inhibition of myosin II recruitment by Tpm3.1/3.2.

Tpm3.1/3.2-mediated coupling of actin filaments to myosin II dimers facilitates actomyosin contractility. Our data, and a previous report (72), show that BCR-induced B-cell spreading does not require myosin or myosin activity. However, when B cells spread across the surface of an APC or lipid bilayer in which antigens are mobile, membrane protrusions are subsequently retracted in a myosin-dependent manner (72). This, together with the myosin-dependent retrograde flow of the peripheral actin (85) and the contractile function of the actomyosin arcs (68), propels the centripetal movement of BCR microclusters and leads to the formation of a cSMAC. Further work is required to determine whether the actin-stabilizing functions of Tpm3.1/3.2, and its ability to couple actin filaments to myosin II, are essential for membrane retraction, BCR microcluster centralization, and cSMAC formation at the immune synapse.

Because actin and actomyosin contractility are essential for cytokinesis (86–89), we tested whether targeting Tpm3.1/3.2 would inhibit the growth of DLBCL cells. Indeed, in 48- to 72-hour assays, ATM-3507 inhibited the growth of NU-DUL-1, TMD8, Toledo, and SU-DHL-8 DLBCL cells with low micromolar or sub-micromolar IC_50_ values. Growth inhibition was accompanied by an increase in both cell size and the percentage of cells with 4N DNA content, suggesting that the ATM-3507-treated cells were arrested in the G2/M phase of the cell cycle and were unable to undergo cytokinesis, as has been observed in neuroblastoma cells (31). Interestingly, myosin IIA-deficient murine B cells also exhibit impaired cytokinesis and accumulate in the G2/M phase of the cell cycle, along with increased cell size (90). Hence, Tpm3.1/3.2 may have a key role in organizing the actomyosin structures that mediate chromosome segregation and cell division. In HeLa cells undergoing mitosis, Tpm3.1 co-localizes with cortical actin filaments and interacts with proteins that link the mitotic spindle to the actin cortex and determine its orientation (56). Further investigation is needed to elucidate how ATM-3507 induces cell cycle arrest in DLBCL cells. Because the ATM-Chk2-p53 pathway initiates apoptosis in tetraploid cells that fail to undergo cytokinesis after DNA replication (91, 92), Tpm3.1/3.2 inhibition may induce apoptosis in p53-positive DLBCL cells.

Our data also indicate that Tpm3.1/3.2 is critical for the migration and motility of DLBCL cells. ATM-3507 treatment significantly reduced the ability of Toledo and NU-DUL-1 DLBCL cells to migrate toward CXCL12, a chemokine that directs the *in vivo* trafficking of both normal and malignant B cells (14, 77). ATM-3507 also reduced the CXCL12-dependent motility of Toledo DLBCL cells on FN, as well as the ability of NU-DUL-1 DLBCL cells to extend leading-edge lamellipodia and assume a motile polarized morphology. Cell motility on FN and other extracellular matrix components supports the movement of extravasated B cells into the underlying tissues. Actin structures and actomyosin contractility are essential for both phases of cell motility, leading-edge protrusion and trailing-edge retraction (93). The ability of ATM-3507 to inhibit the motility and migration of DLBCL cells may reflect crucial roles for both the actin-stabilizing and actin-myosin coupling functions of Tpm3.1/3.2 in these processes. Notably, murine B cells lacking myosin IIA exhibit impaired migration and defective *in vivo* trafficking (90). By impairing chemokine-induced DLBCL cell motility, ATM-3507 could limit the ability of DLBCL cells to migrate into tissues and establish extranodal sites of tumor growth.

Although it remains to be confirmed by *in vivo* preclinical studies, the ability of ATM-3507 to limit the *in vitro* growth and motility of DLBCL lines suggests that Tpm3.1/3.2 could be a therapeutic target for inhibiting DLBCL progression and dissemination. The utility of ATM-3507 as a potential cancer therapy depends on the balance between its efficacy and toxicity, which could be due to off-target effects. Although phase 1 clinical trials of ATM-3507 (Anisina) for the treatment of neuroblastoma were terminated due to toxicity (94), the development of more potent and selective Tpm3.1/3.2 inhibitors is being pursued. Importantly, *in vitro* tests of drug combinations, especially drugs targeting different cellular processes or cancer cell vulnerabilities, often reveal synergistic effects that enable the use of substantially lower drug concentrations. Indeed, ATM-3507 synergizes with low concentrations of the microtubule-disrupting drug vincristine, a component of R-CHOP, to block mitosis and induce apoptosis in various tumor cell lines (31, 56, 75). Pairing the actin-directed effects of ATM-3507 with the microtubule-targeting drug vincristine, in the context of the R-CHOP, is the most likely manner in which anti-tropomyosin drugs could be used to increase the efficacy of DLBCL treatment regimens. A recent study demonstrated multi-component synergistic killing of DLBCL cell lines by various combinations of ibrutinb (Btk inhibitor), Venetoclax (Bcl2 inhibitor), lenalidomide (promotes degradation of the IKZF1 and IKZF3 transcription factors that act downstream of the NF-κB/IRF4 pathway to promote the survival of malignant B cells), and prednisone (an R-CHOP component that inhibits the NF-κB pathway) (95). The ability of low doses of Tpm3.1/3.2 inhibitors to enhance the efficacy of R-CHOP components, immune checkpoint inhibitors, or drugs targeting pathways that are crucial for B-cell survival (e.g., ibrutinib, Syk inhibitors, phosphoinositide 3-kinase inhibitors) should be investigated. *In vitro* studies of drug combination efficacy would lay the groundwork for pre-clinical studies in mice using DLBCL cell lines or patient-derived xenografts.

By using the Tpm3.1/3.2-selective inhibitor ATM-3507, we provide the first evidence that tropomyosin regulates B-cell actin dynamics. Whether other Tpm isoforms modulate actin dynamics in B cells and have distinct functions remains to be determined. However, our data suggest that Tpm3.1/3.2 have non-redundant functions in B cells. The structure of ATM-3507 has been optimized to target a unique C-terminal pocket in Tpm3.1 and Tpm3.2 that is encoded by exon 9d of the *Tpm3* gene. Nevertheless, further studies in which *Tpm3* exon 9d is selectively deleted in B cells would provide important confirmation regarding the role of Tpm3.1/3.2 in B cells and rule out potential off-target effects of ATM-3507. In that regard, Kee et al. (38) showed that ATM-1001, a structural analogue of ATM-3507 that binds to the exon 9d pocket in a similar manner as ATM-3507 (29), inhibits glucose uptake and insulin secretion when injected into wild-type mice but not knockout mice in which *Tpm3* exon 9d had been deleted. These findings argue against off-target effects of these anti-tropomyosin drugs. Finally, Tpm3.1/3.2 has been suggested as a target for cancer therapy, and we showed that ATM-3507 effectively inhibits the growth and motility of DLBCL cells. Further work could investigate its efficacy for other types of B-cell malignancies.

## Data Availability

The raw data supporting the conclusions of this article will be made available by the authors, without undue reservation.
