# Supplementary material for: The tropomyosin 3.1/3.2 inhibitor ATM-3507 alters B-cell actin dynamics and impairs the growth and motility of diffuse large B-cell lymphoma cell lines
Source: Front Immunol. 2025 Nov 5;16:1668379. doi: 10.3389/fimmu.2025.1668379 (PMC12626929; doi:10.3389/fimmu.2025.1668379)
Supplement: Supplementary file 1 [file DataSheet1.pdf]

Supplementary Material

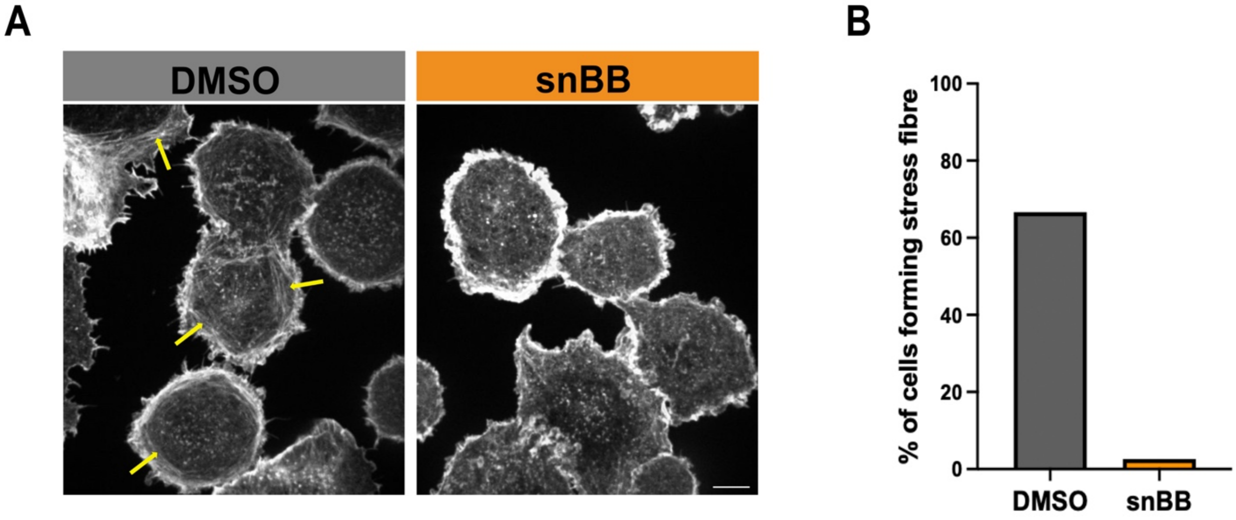

Supplementary Figure 1. snBB treatment inhibits stress fiber formation during cell spreading in B16-F1 murine melanoma cells. (**A**) B16-F1 murine melanoma cells were pre-treated with either 50 µM snBB or an equivalent volume of DMSO (0.5% final concentration) for 1 h at 37°C. The cells were added to FN-coated coverslips and allowed to spread for 30 min before being stained with rhodamine-phalloidin to visualize F-actin. (**A**) Representative confocal microscopy images. Yellow arrows point to stress fibers. Scale bar is 10 µm). (**B**) Percent of cells that formed stress fibers. n >30 cells per condition.

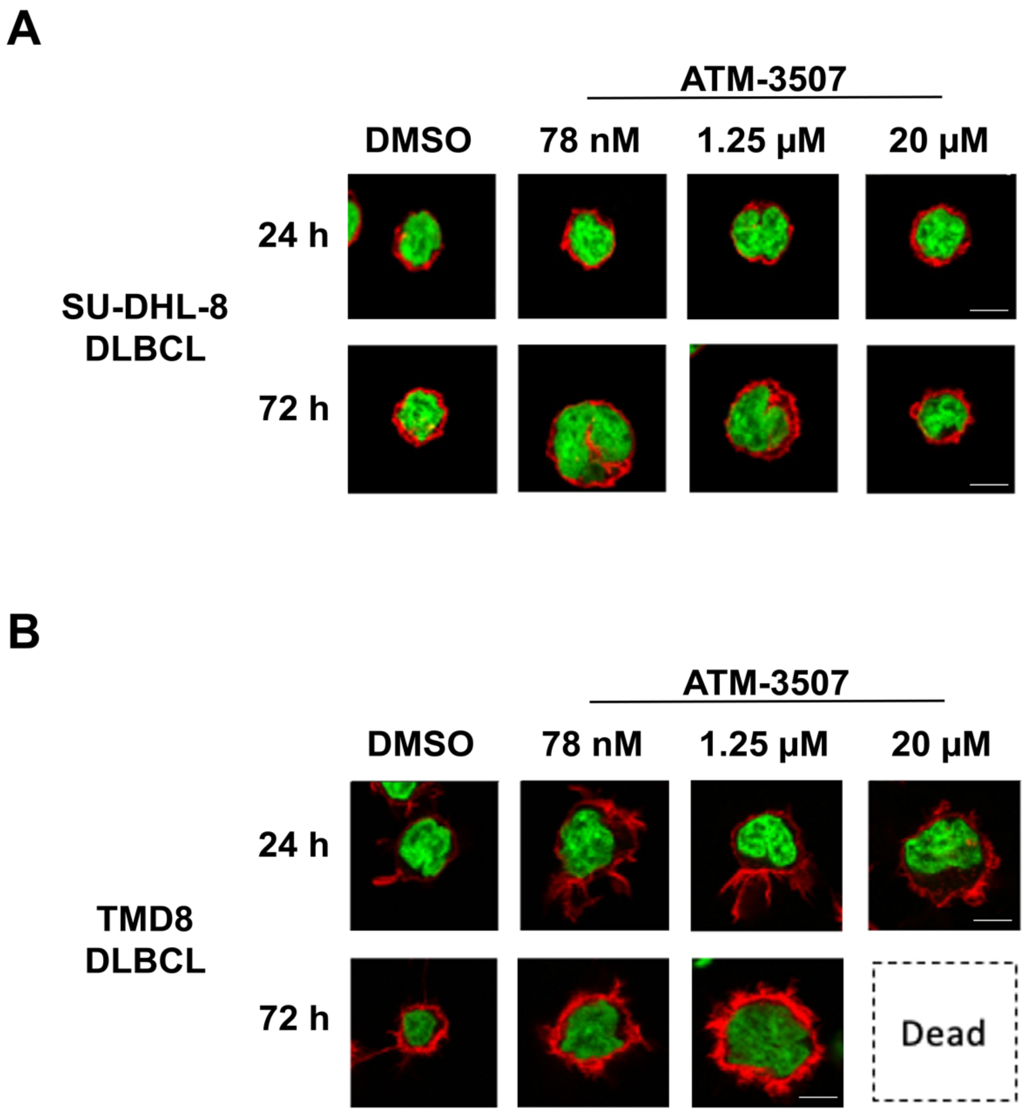

Supplementary Figure 2. ATM-3507 treatment of DLBCL cells increases cell size and causes nuclear enlargement. (**A**) SU-DHL-8 or TMD8 DLBCL cells were cultured with 0.03% DMSO or the indicated concentrations of ATM-3507 for 24 h or 72 h before being stained with rhodamine-phalloidin and DAPI. Cells were imaged by confocal microscopy. Representative images are shown.

Captions for videos

Video 1. Toledo DLBCL cells were added to FN-coated coverslips in the presence of 100 nM CXCL12 and 0.1% DMSO. After a 1 h pre-treatment period, time-lapse images were acquired every 30 s for 1 h. The video playback rate is 10 frames per second. Each second of the video represents 5 minutes of real time (300X real speed). Similar results were obtained in 3 independent experiments. Videos 1 and 2 are DMSO (control) and ATM-3507-treated cells from the same experiment.

Video 2. Toledo DLBCL cells were added to FN-coated coverslips in the presence of 100 nM CXCL12 and 10 μM ATM-3507. After a 1 h pre-treatment period, time-lapse images were acquired every 30 s for 1 h. The video playback rate is 10 frames per second. Each second of the video represents 5 minutes of real time (300X real speed). Similar results were obtained in 3 independent experiments. Videos 1 and 2 are DMSO (control) and ATM-3507-treated cells from the same experiment.

Video 3. NU-DUL-1 DLBCL cells were added to FN-coated coverslips in the presence of 100 nM CXCL12 and 0.1% DMSO. After a 1 h pre-treatment period, time-lapse images were acquired every 30 s for 1 h. The video playback rate is 10 frames per second. Each second of the video represents 5 minutes of real time (300X real speed). The percent of cells that developed a polarized morphology at any time during the observation period was determined visually. Similar results were obtained in 5 independent experiments. Videos 3 and 4 are DMSO (control) and ATM-3507-treated cells from the same experiment.

Video 4. NU-DUL-1 DLBCL cells were added to FN-coated coverslips in the presence of 100 nM CXCL12 and 10 μM ATM-3507. After a 1 h pre-treatment period, time-lapse images were acquired every 30 s for 1 h. The video playback rate is 10 frames per second. Each second of the video represents 5 minutes of real time (300X real speed). The percent of cells that developed a polarized morphology at any time during the observation period was determined visually. Similar results were obtained in 4 independent experiments. Videos 3 and 4 are DMSO (control) and ATM-3507-treated cells from the same experiment.
